# Supplementary material for: The Evolution and Characterization of the RNA Interference Pathways in Lophotrochozoa
Source: Genome Biol Evol. 2024 May 7;16(5):evae098. doi: 10.1093/gbe/evae098 (PMC11114477; doi:10.1093/gbe/evae098)
Supplement: evae098_Supplementary_Data [file evae098_supplementary_data.zip › Supplementary_materials.zip!/selected_miRNAs/NoGe_selected_miRNAs/mirdeep_pdf_files/NMRB01000004.1_Notospermus_geniculatus_strain_Ushimado_scaffold4,_whole_genome_shotgun_sequence_171.pdf]

Provisional ID	:	NMRB01000004.1_Notospermus_geniculatus_strain_Ushimado_scaffold4,_whole_genome_shotgun_sequence_171
Score total	:	603.2
Score for star read(s)	:	3.9
Score for read counts	:	598.7
Score for mfe	:	1.2
Score for randfold	:	0
Score for cons. seed	:	-0.6
Total read count	:	1186
Mature read count	:	1171
Loop read count	:	0
Star read count	:	15

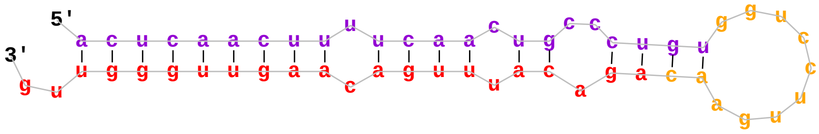

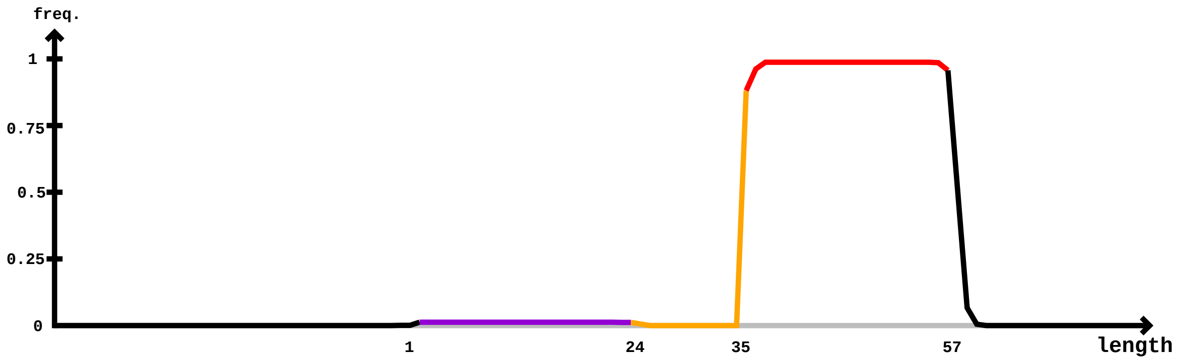

Star **Mature**

```
5'- gaagagacagugacuggacuggaguugguaauaucgacucaacuuuucaacugcccuguggguccuugaacagacauuugacaaguuggguugauguucacaaaccaagcauu   -3'    obs
    gaagagacagugacuggacuggaguugguaauaucgacucaacuuuucaacugcccuguggguccuugaacagacauuugacaaguuggguugauguucacaaaccaagcauu          exp
    .......((((......))))...(((((((((((((((((((((.((((.((..((((.........)))).)).)))).))))))))))))))))).....)))))..... reads   mm   sample
    ......................................................................agacauuugacaaguuggguCg.................... 1       1    Fm1
    ......................................................................aAacauuugacaaguuggguug.................... 1       1    Fm1
    ......................................................................agacauuugacaaguuggguGg.................... 2       1    Fm1
    ......................................................................agacauuugacaaguugCguug.................... 1       1    Fm1
    ......................................................................agacauuugacaaguuggguug.................... 62      0    Fm1
    ......................................................................agacauuugacaaguuCgguug.................... 1       1    Fm1
    ......................................................................agacauuugacaaguuggguuga................... 11      0    Fm1
    ......................................................................agacauuugacaaguuggguugU................... 70      1    Fm1
    ......................................................................agacauuugacaaguuggguugG................... 1       1    Fm1
    ......................................................................agacauuugacaaguuggguugaA.................. 2       1    Fm1
    ......................................................................agacauuugacaaguuggguugUu.................. 4       1    Fm1
    ......................................................................agacauuugacaaguuggguugau.................. 3       0    Fm1
    .......................................................................gacauuugacaaguuggguuga................... 8       0    Fm1
    ........................................................................acauuugacaaguugggCugau.................. 1       1    Fm1
    ........................................................................acauuugacaaguuggguugau.................. 4       0    Fm1
    ........................................................................acauuugacaaguuggguugauA................. 5       1    Fm1

    ......................................................................agacauuugacaaUuggguu...................... 1       1    Fm2
    ......................................................................agacauuugacaaguuggguu..................... 14      0    Fm2
    ......................................................................agacauuugacaaguuggguGg.................... 1       1    Fm2
    ......................................................................agacauuugacaaguuggguug.................... 180     0    Fm2
    ......................................................................agacauuugacaaguugCguug.................... 3       1    Fm2
    ......................................................................agacauuugacaGguuggguug.................... 4       1    Fm2
    ......................................................................agacauuugacaaguugAguug.................... 2       1    Fm2
    ......................................................................agacauuugacaaguuggguugU................... 94      1    Fm2
    ......................................................................agacauuugacaaguuggguuga................... 50      0    Fm2
    ......................................................................agacauuugacaGguuggguuga................... 4       1    Fm2
    ......................................................................agacauuugacaaguuggguugG................... 2       1    Fm2
    ......................................................................agacGuuugacaaguuggguuga................... 1       1    Fm2
    ......................................................................agacauuugacaaguuggguugau.................. 5       0    Fm2
    ......................................................................agacauuugacaaguuggguugUu.................. 8       1    Fm2
    .......................................................................gacauuugacaaguuggguug.................... 6       0    Fm2
    .......................................................................gacauuugacaaguuggAuuga................... 1       1    Fm2
```

Star Mature

```
gaagagacagugacuggacuggaguugguaauaucgacucaacuuuucaacugcccuguggucuugaacagacauuugacaaguuggguugauguucacaaaccaagcauu
......................................................................gacauuugacaaguuggguuga...................   41   0   Fm2
.......................................................................acauuugacaaguuggguugaA..................   2    1   Fm2
.......................................................................acauuugacaaguuggguugau..................   9    0   Fm2

.....................................................................agacauuugacaaguugggu......................   1    0   Mm1
.....................................................................agacauuugacaaguuggguug....................   14   0   Mm1
.....................................................................agacauuugacaaguuggguugU...................   24   1   Mm1
.....................................................................agacauuugacaaguuggguugUu..................   3    1   Mm1

..................................cgacucaacuuuucaacugcccG......................................................   1    1   Mm2
....................................acucaacuuuucaacugcccugu....................................................   9    0   Mm2
.....................................................................agacauuugacaaguugggu......................   1    0   Mm2
.....................................................................agacauuugacaaguuggguu.....................   6    0   Mm2
.....................................................................agacauuugaUaaguuggguug....................   2    1   Mm2
.....................................................................agacauuugacaaguuUgguug....................   1    1   Mm2
.....................................................................agacauuugCcaaguuggguug....................   1    1   Mm2
.....................................................................agacauuugacaaguuggguuU....................   1    1   Mm2
.....................................................................agacauuugacaaguugAguug....................   1    1   Mm2
.....................................................................agacauuugacaaguuggguug....................   62   0   Mm2
.....................................................................agacauuugacaaguCggguug....................   1    1   Mm2
.....................................................................agacauuugacaaguuggguuga...................   22   0   Mm2
.....................................................................agacauuugacaaguuggguugU...................   170  1   Mm2
.....................................................................agacauuugacaaguuggguugG...................   2    1   Mm2
.....................................................................agacauuugacaaguuggguugau..................   2    0   Mm2
.....................................................................agacauuugacaaguuggguugUu..................   6    1   Mm2
.....................................................................agacauuugacaaguuggguugaG..................   1    1   Mm2
......................................................................gacauuugacaaguuggguuga...................   13   0   Mm2
.......................................................................acauuugacaaguuggguuga...................   1    0   Mm2
.......................................................................acauuugacaaguuggguugU...................   1    1   Mm2
.......................................................................acauuugacaaguuggguugau..................   6    0   Mm2
.......................................................................acauuugacaaguuggguugauU.................   1    1   Mm2

....................................acucaacuuuucaacugcccugu....................................................   1    0   Fm3
.....................................................................agacauuugacaaguuggguu.....................   10   0   Fm3
.....................................................................agacauuugacaaguugUguu.....................   1    1   Fm3
.....................................................................agacauuugacaaguuggguuU....................   1    1   Fm3
.....................................................................agacauuugacaaguuggguug....................   54   0   Fm3
.....................................................................agacauuugacaaguuggguuga...................   18   0   Fm3
.....................................................................agacauuugacaaguuggAuuga...................   1    1   Fm3
.....................................................................agacauuugacaaguuggguugU...................   41   1   Fm3
.....................................................................agacauuugacaaguuggguugau..................   2    0   Fm3
......................................................................gacauuugacaaguuggguug....................   2    0   Fm3
......................................................................gacauuugacaaguuggguuga...................   14   0   Fm3
......................................................................gacauuugacaaguuggguugU...................   2    1   Fm3
......................................................................gacauuugacaaguuggguugau..................   3    0   Fm3
......................................................................gacauuugacaaguuggguugaA..................   6    1   Fm3

....................................acucaacuuuucaacugcccugu....................................................   4    0   Mm3
.....................................................................agacauuugacaaguuggguu.....................   2    0   Mm3
.....................................................................agacauuugacaaguuggguuU....................   1    1   Mm3
.....................................................................agacauuugacaaguuggguug....................   10   0   Mm3
.....................................................................agacauuugacaaguuggguuC....................   1    1   Mm3
.....................................................................agacauuugacaaguuggguuga...................   3    0   Mm3
.....................................................................agacauuugacaaguuggguugU...................   45   1   Mm3
.....................................................................agacauuugacaaguuggguugau..................   2    0   Mm3
.....................................................................agacauuugacaaguuggguugUu..................   3    1   Mm3
.....................................................................agacauuugacaaguuggguugaA..................   1    1   Mm3
```
